# Factors influencing the elderly’s behavioural intention to use smart home technologies in Saudi Arabia

**DOI:** 10.1371/journal.pone.0272525

**Published:** 2022-08-30

**Authors:** Kholoud Maswadi, Norjihan Abdul Ghani, Suraya Hamid

**Affiliations:** 1 Faculty of Computer Science and Information Technology, University Malaya, Kuala Lumpur, Malaysia; 2 Department of Management Information Systems, Jazan University, Jazan, Saudi Arabia; TDTU: Ton Duc Thang University, VIET NAM

## Abstract

In recent years, smart home technologies have offered opportunities for elderly people to manage their daily health-related activities. Despite the advancement in smart home technology (SHT), the level of end-user acceptance among elderly people is still low. This study proposes an SHT framework by examining the determinants of elderly behavioural intention (BI) to use smart home technologies by extending the Unified Theory of Acceptance and Use of Technology (UTAUT) model. This study uses the quantitative approach to survey about 486 elderly people in Saudi Arabia, and it applies the Partial Least Square Structural Equation Model (PLS-SEM) technique to perform the data analysis. Findings reveal that culture influence and technology awareness are significant factors in determining the BI to use SHT among elderly people. The study also finds that attitude mediates the relationships between performance expectancy, effort expectancy, and behavioural intention. We find that region and education moderate the relationships between culture influence, technology awareness, and behavioural intention. This study theoretically extends the UTAUT theory by including external constructs: culture influence, technology awareness, attitude, education, and region.

## 1. Introduction

Nowadays, the Internet of Things (IoT) plays a fundamental role in ensuring a better, easier, and more comfortable quality of life. Many companies including Google, and Apple are increasingly introducing the IoT technologies to improve our lives [1], suggesting that IoT applications such as smart home technologies (SHTs) are provided to aid various aspects of the daily activities of users relating to healthcare, traffic monitoring, water supply, and energy saving. Recently, SHTs offer a range of services that are remotely controlled and linked to a network system to provide human independence and hence, lead to enhanced quality of life for the elderly [2].

A smart home is described as “a residence equipped with a high-tech network, linking sensors and domestic devices, appliances, and features that can be remotely monitored, accessed or controlled, and provide services that respond to the needs of its inhabitants” [3]. Meanwhile, SHTs for elderly people can be described as the “information-based technology that passively collects and shares elderly residents’ information with the elderly themselves and family members in addition to primary care providers” [4]. Several smart home technologies such as Google Nest are available for the elderly to help them monitor their activities daily to be healthy while living independently. Recent statistics show that the number of SHTs is estimated to increase from 7.8M in 2020 to 10.7M in 2024 [3]. SHTs ensure the security and comfort of elderly people by allowing them to control smart devices (e.g., smart lighting, smart locks, temperature sensors, etc.) using applications on their smartphones, voice commands, etc. For instance, the use of an automatic lighting system allows the control of lights when the elderly enter or exit a room, which reduces energy consumption.

Globally, the number of the elderly is increasing. For instance, in Saudi Arabia, according to the survey conducted in 2017 by the General Authority of Statistics [5], the number of elderly people (60+ years) constitutes about 4.19% of the total Saudi population. This number is estimated to reach about 18% of the total Saudi population by the year 2050 [5]. Meanwhile, the ageing society has posed serious challenges to the limited number of healthcare facilities available and the elderly’s quality of life. Statistics show that about an 8.5million older adults of 60 years need daily assistance and care, with about 80% of seniors over 65 years living with at least one chronic disease [6]. Thus, several reasons account for the need for caregivers for the elderly such as enhanced mobility, and emotional support. In the United States, around 39.8 million caregivers provide healthcare services for adults. Therefore, smart devices (e.g., smart fridges, smart lights, and smart fire alarm systems) facilitate the elderly’s daily activities, ensure the independence of elderly people, and improve their well-being. However, despite the benefits of smart technologies for the elderly, they are still not very much known.

Researchers in technology management have posited that the intention to use a certain technology is relevant and a strong determinant and predictor of the actual use of the technology, which in turn, predicts users’ future usage. Therefore, one of the major concepts in technology acceptance models is the behavioural intention to use [7–9]. However, the level of SHTs acceptance among elderly people in developing countries including Saudi Arabia is very low [10, 11]. Prior studies seemed to have focused much attention on the technological aspects of SHTs with less attention on the behavioural aspects [12, 13]. Although a few researchers have examined the drivers of SHTs intention to use by considering different factors like technology trust, security risk, and cost [14, 15], many crucial factors especially different contextual factors are yet to be explored and investigated.

In Saudi Arabia and other Arab countries, a lack of examination of contextual factors such as cultural influence and technology awareness may affect the variations in behaviour intention to use SHT among elderly people. The Arab society seems to be inclined to a defined cultural norm and values that shape the belief systems, activities, businesses, and the total way of life of its people. Hence, the use of any technology is a function of cultural acceptance and leadership backing [16]. Past studies on SHTs did not examine these pertinent contextual factors and that explains the reasons why the level of SHTs usage is still very low in Saudi Arabia. Therefore, the need to further examine contextual factors and other demographic factors that could affect the elderly’s intention to use SHTs.

Motivated by the suggestions of [10, 12, 17] that future research should examine the Arab contextual factors, this study extends our understanding of the impact of culture influence, technology awareness, and other demographic factors on people’s intention to use SHTs. Statistics show that more than 90% of elderly people live independently and about 85% of them prefer to receive their health treatments in their homes provided that important health facilities are available [13, 18]. Therefore, SHT could enhance the quality of life of elderly people [12], monitor and manage their daily health conditions [19], and reduce the cost of elderly’s health care and caregivers’ time [12]. Given this significance, it appears that elderly people must be ready to relate with their health-related technologies to improve their health conditions. [20] noted that the success of any new technology is to understand the relationships between the technology and the users of it.

In this paper, we examine the contextual predictors of BI to use SHTs among elderly people. Further, we investigate the moderating role of region and education on the relationships between culture influence, technology awareness and behavioural intention to use SHTs. Understanding the reasons behind users’ acceptance or refusal of smart home services is relevant for the success of any new technologies and will certainly increase their adoption, making them more attractive, and interesting to use. Hence, it is important to identify motivating factors as well as issues that affect the elderly behavioural intention toward SHTs. Recent research studies have successfully applied technology acceptance theories (e.g., Technology Acceptance Model—TAM, and UTAUT) to understand the elderly’s intention toward smart technologies [21, 22].

The contribution of this study is three-fold. First, we extend the theoretical prediction of the UTAUT model in explaining BI to use SHTs using two contextual factors: culture influence and technology awareness. Our findings show that both variables have positive and significant impacts on behavioural intention. Second, by integrating attitude construct from the TAM into the UTAUT theory, this study further contributes to the prediction of behavioural intention that effort expectancy and performance expectancy could also have indirect effects on behavioural intention to use SHTs. Third, unlike past studies that used age and income groups as moderators in the UTAUT model, this study introduces a new contextual moderator, region to a potential moderator. We find that region along with education moderate the relationships between culture influence, technology awareness, and BI to use SHTs.

The rest of the paper is organized as follows. Section 2 provides the background review of some related work. Section 3 provides the theoretical underpinnings and hypotheses development. The methodology used in this paper is provided in section 4. Section 5 presents the discussion of the findings while section 6 concludes the paper.

## 2. Background and related work

Here, we present the background information about SHT services, their benefits for the elderly, and the related technology acceptance theories.

### 2.1 Smart home technologies and services for the elderly

**Fig 1:**
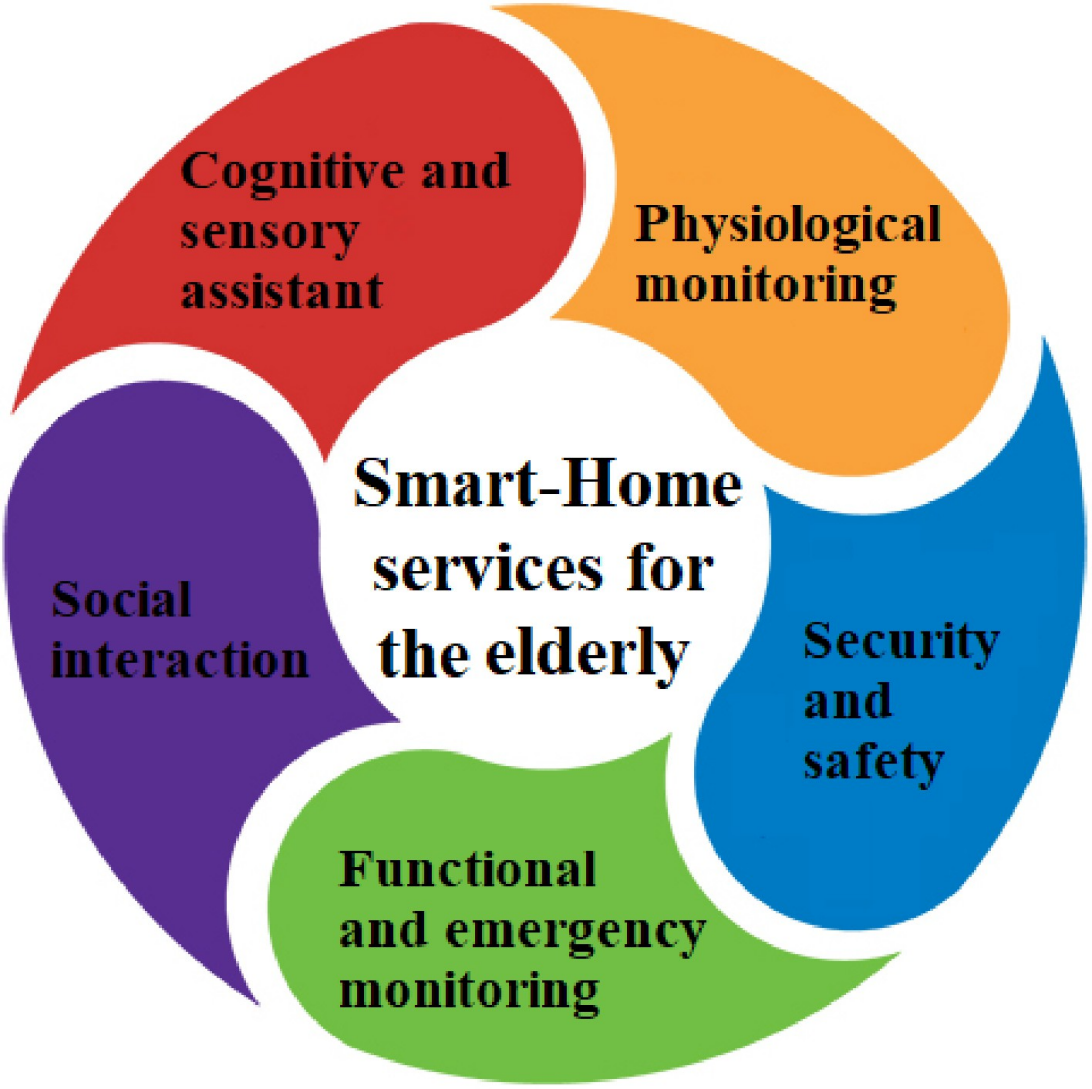
Smart-home technologies and services for the elderly.

A smart home is an innovative home where different smart devices can be controlled by the resident through smartphones, tablets, etc. Worldwide, there are 175 million smart homes, this number is estimated to increase by 25% from 2020 to 2025 [23]. Smart homes allow various services that facilitate daily duties and enhance the quality of our lives. SHTs are increasingly being adopted by the elderly mainly for healthcare and social support [24]. The survey conducted by Wilson et al., 2017 showed that SHTs are mainly used to ensure convenience and better security and to enhance entertainment and communication in homes. Fig 1 presents the five categories of SHT: Physiological monitoring, security and safety assistance, functional and emergency monitoring, social interaction, and cognitive and sensory assistance.

#### 2.1.1 Physiological monitoring technologies

Controlling and measuring the relevant physiological parameters for the elderly is important to maintain a healthy independent life as much as possible and avoid any complications. However, as a person gets older, he may experience some problems with his memory and other thinking abilities. Temperature, blood pressure, respiratory rate, heartbeat rate, and blood oxygen saturation are the main five medical signs for the elderly as documented by scientists [25]. In this context of SHTs, smart homes offer many devices, especially for people who endure chronic diseases (e.g., diabetes, asthma, etc.) to help them be more independent. Smart home services can help the elderly to remember daily tasks such as taking medicine and blood glucose [26].

#### 2.1.2 Security and safety assistance technologies

According to World Health Organization (2021), one in six older adults is potentially suffering from abuse either physically, mentally, or financially. Gerontologists consider that the elderly’s safety and security have a significant influence on their self-confidence. The sense of insecurity for older adults may be caused by many factors such as loneliness, environment, health issues, etc. This sense can be more important for isolated elderly or with disabilities. Smart homes provide several security devices that can be used in supervising houses, locking or unlocking doors and windows, smart smoke alarms, etc. Smart security devices can be controlled by voice command or using smartphones, which make their use easy for the elderly. For example, Smart Life Technology Company in Saudi Arabia provides security cameras to monitor any intruder entering the protected areas. Whenever an intruder enters the home, the alarm in the security camera starts sounding loud, and the screen automatically starts showing the intruder. A notification is automatically received on one’s mobile device and all lights are automatically turned on making it easier to handle the security situation.

#### 2.1.3 Functional and emergency monitoring technologies

Emergency services provided by smart homes play a vital role in the elderly lives. Whenever there is an abnormal situation, smart home emergence services systems can automatically send emergency alerts/calls to hospitals, family members, fire departments, or others [27]. As an example, the elderly GPS smart watch for people with Alzheimer’s allows a quick localization, and it can automatically send an alert to the caregivers or family members when the elderly leave a safe zone. On the other hand, older adults may have some disabilities and mobility issues. For this reason, they may need help in controlling home functions. In smart homes, home functions can be managed from a distance through remote control, and voice command. For instance, a smart oven, smart lights, smart heater and so on can be controlled from a distance with a remote control or a smartphone. For example, Nintendo Co.’s new brain-training game for the elderly, ’Brain Age’ (known in Japan as brain training). The console is a collection of cognitive exercises designed for those over the age of 45 that claim to increase mental agility and even delay the onset of dementia and Alzheimer’s disease [28].

#### 2.1.4 Social interaction technologies

Elderly people often suffer from loneliness, which affects their mental health. Smart robots are deployed as social companions for the old and socially isolated people. They have been successfully used by people who are suffering or recovering from an illness. Moreover, smart robots are very helpful for people with dementia to change the elderly’s moods and reduce their feeling of isolation and boredom [24]. Another smart speaker device: “Google Nest” (or Google Home) can be helpful for the elderly to enhance their social interaction (named previously Google Home). This device allows the elderly to speak voice commands to control and manage lights, TVs, and others. Human android robots have been introduced by Aldebaran Robotics (France) named ‘Nao’ which is responsive to voice, eye gaze, and gesticulation [29]. Service-type robots support the basic activities (e.g., eating, bathing, using the toilet, and getting dressed), enhance the mobility level, provide household maintenance, and monitor individuals that need permanent attention. Examples of these types of robots are the nursebot Pearl, the Dutch iCat, and the German Care-obot [30].

#### 2.1.5 Cognitive and sensory assistant technologies

Several environmental control systems such as smart heating can reduce energy consumption. Smart home statistics show that smart heating can save users 50% in energy use [23]. The intelligent personal assistant “Alexa,” developed by Amazon Lab 126, has been installed in a wide range of products. LG Electronics has adopted Alexa throughout its smart home product lines. For example, if a user calls “Alexa” from a smart refrigerator, the user can access its services. Also, a smart lighting system can control the light by switching it on and off depending on whether the owner is in the room or not. This information can be provided using sensors, and smartphones.

### 2.2 Benefits of smart homes

Many challenges may affect older adults’ lifestyles such as physical limitations (e.g., balance, reaching, etc.), perceptual feelings (e.g., vision, hearing, etc.), and cognitive behaviour (e.g., Memory, parallel tasks, etc.). Hence, older people may need help with their daily activities such as transportation, memory functioning, and health monitoring, and can be achieved using smart home devices.

**Fig 2:**
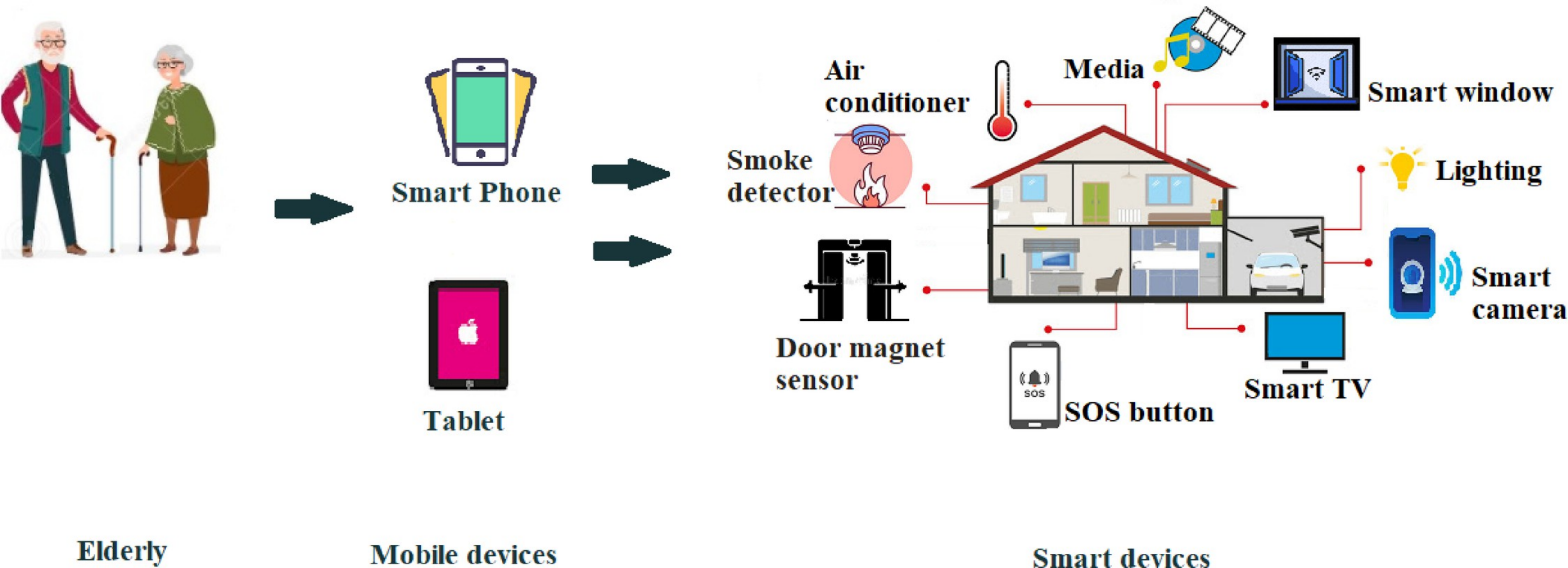
A set of smart home devices.

As shown in Fig 2, mobile devices (e.g., smartphones, tablets, etc.) can be used to control several smart home devices—smart TV, smart camera, and lighting. Despite the advantages of smart home services for older adults, the older generation is less familiar with new smart technologies [31]. The benefits of smart homes include independent living, improved healthcare, social involvement, safety, cost reduction, and decision making as discussed as follows.

#### 2.2.1 Independent living

The main benefit of smart homes for the elderly is to ensure their independence, if possible, especially for people with disabilities or mobility problems. The fact that each smart device can be controlled from a distance using smartphones, tablets or smart speakers reduces the senior’s movement. Hence, SHTs make it easier for the elderly to live independently without the need for a caregiver’s assistance.

For example, people with dementia or Alzheimer’s may forget to turn off the stove- tops and ovens, which is very dangerous for them. Smart devices (e.g., smart ovens, smoke detectors, etc.) can detect abnormal situations, and some systems can automatically turn off the appliances. Moreover, smart thermostats can adjust the temperature by heating or cooling the home automatically, which can be beneficial in energy saving. Even making phone calls can be easier using smart technologies from anywhere around the house. This enables the elderly to communicate with their relatives, friends, and doctors in a convenient and timely manner. [12].

#### 2.2.2 Improving healthcare

As a person get older, he/she may have some health issues. The common health problems that may appear due to ageing are chronic diseases, mental disorders, mobility problems, malnutrition, and others. In this context, smart home devices can help the elderly monitor their health and avoid any complications. For instance, wearable biomedical sensors help to track health-related issues including blood pressure, blood sugar, etc. Some smart home systems can send automatically emergency alerts to elderly caregivers to help them react quickly. The main problem with chronic diseases is that they can be risky if the patient forgets to take the medication at the right time and with the right amounts. For this reason, a smart assistant routine can remind the elderly to take medications as prescribed, to measure blood glucose levels [31].

#### 2.2.3 Assisting with daily activities

Often, older people have certain trouble performing some daily tasks (e.g., cleaning, shopping, etc.), especially people with mobility issues. Some simple daily activities, such as grocery shopping, can be problematic for seniors. Smart home devices can help the elderly with daily activities. For instance, smart refrigerators can detect the quality of food left in the fridge and inform the homeowner when he needs to do grocery shopping. Some smart assistants can also automatically order the needed items online so that the groceries can be delivered to the home.

On the other hand, automated doors and drawers can also make the navigation around the elderly homes easier, comfortable and ensure their safety. Moreover, robot vacuums help clean the house without too much effort needed [32].

#### 2.2.4 Social involvement

Social isolation and loneliness impact negatively older adults, especially those with disabilities or mobility issues. In addition, recent studies demonstrated that the COVID-19 pandemic and the social distancing guidelines have restricted social activities and impacted older adults [27]. Smart home technology provides communication devices to help the elderly easily contact their families and friends such as visual communication systems and capturing facial expressions [19, 33]. Furthermore, enhancing the relationships with others and allowing follow-up the social activities which increase the self-esteem of these elderly people.

#### 2.2.5 Safety

The security and safety of older adults are a big issue, especially when they are living alone. The elderly people are an attractive target for criminals. For this reason, smart home devices can be used to ensure home safety and protect the elderly from dangers. For instance, the elderly can control home locks, windows, etc. some sensors can detect any unusual movement inside houses. In case of any danger or security issue, alert notifications can be sent to inform the homeowner, police stations, etc. Another smart service that helps ensure the senior’s safety is the personal emergency response system which is an emergency portable button that can be used when the elderly feel unsafe.

For people with dementia or Alzheimer’s disease, other smart services are provided to keep them safe as much as possible. For example, geofencing allows using GPS to track those people and inform their caregivers if they leave the safe zones through smartphones [34].

#### 2.2.6 Cost reduction

Same vital activities can be costly for the elderly (e.g., medical services, shopping, etc.). For instance, older people living outside of the city may have some mobility difficulties, which make physical visits to hospitals challenging and expensive.

Smart home devices can help the elderly save money, by reducing for example the number of doctor visits. Some studies confirmed that smart homes provide cost-effective home care for older and vulnerable people [19, 35]. Thus, smart devices reduce the cost of basic needs and activities (e.g., medical, security, energy conservation, etc.). For example, the smart security system can reduce energy consumption since it avoids troubles that can be caused by any broken machine [36].

### 2.3 Related works

Smart home technologies assist older people to enhance the quality of their life. However, the acceptance rate of smart homes among the elderly is pretty much low. Meanwhile, studies have established that there is a gap between the needs of elderly people and the services provided [37, 38]. As such, many researchers have investigated the elderly’s acceptance of SHTs by adopting technology acceptance theories (e.g., UTAUT and TAM).

Davis [39] developed the TAM, and today, it is one of the main theories used in technology adoption studies. Perceived usefulness and perceived ease of use serve as the foundational predictors of intention to accept new technologies in TAM. Davis defines perceived usefulness as “the degree to which a person believes that using a particular system would enhance his or her job performance” and perceived ease of use as “the degree to which a person believes that using a particular system would be free of effort”. The more people feel and have a positive attitude towards new technology, the greater the perceived usefulness and perceived ease of use of such technology. Such positive feelings and attitudes increase people’s intention to adopt and use it [3, 40]. Thus, the TAM has been validated and widely used in technology adoption research [14]. TAM has received much attention from different researchers through modifications and extensions from other theories that have appeared in the technology management literature [41–45].

Moreover, Venkatesh et al. [7] developed (UTAUT) based on a comparison of eight models, including the TAM, TRA, TPB, Innovation Diffusion Theory (IDT), the motivational model (MM), a model combining the TAM and TPB, the model of PC application, and the social cognitive theory (SCT). Given the wide, unstructured use and adoption of earlier models, the goal of designing UTAUT was to create a unified perspective of technology adoption [46]. Originally, UTAUT has four constructs which are performance expectancy, effort expectancy, social influence, and facilitating conditions with four key moderators (gender, age, experience, and voluntariness of use).

**Fig 3:**
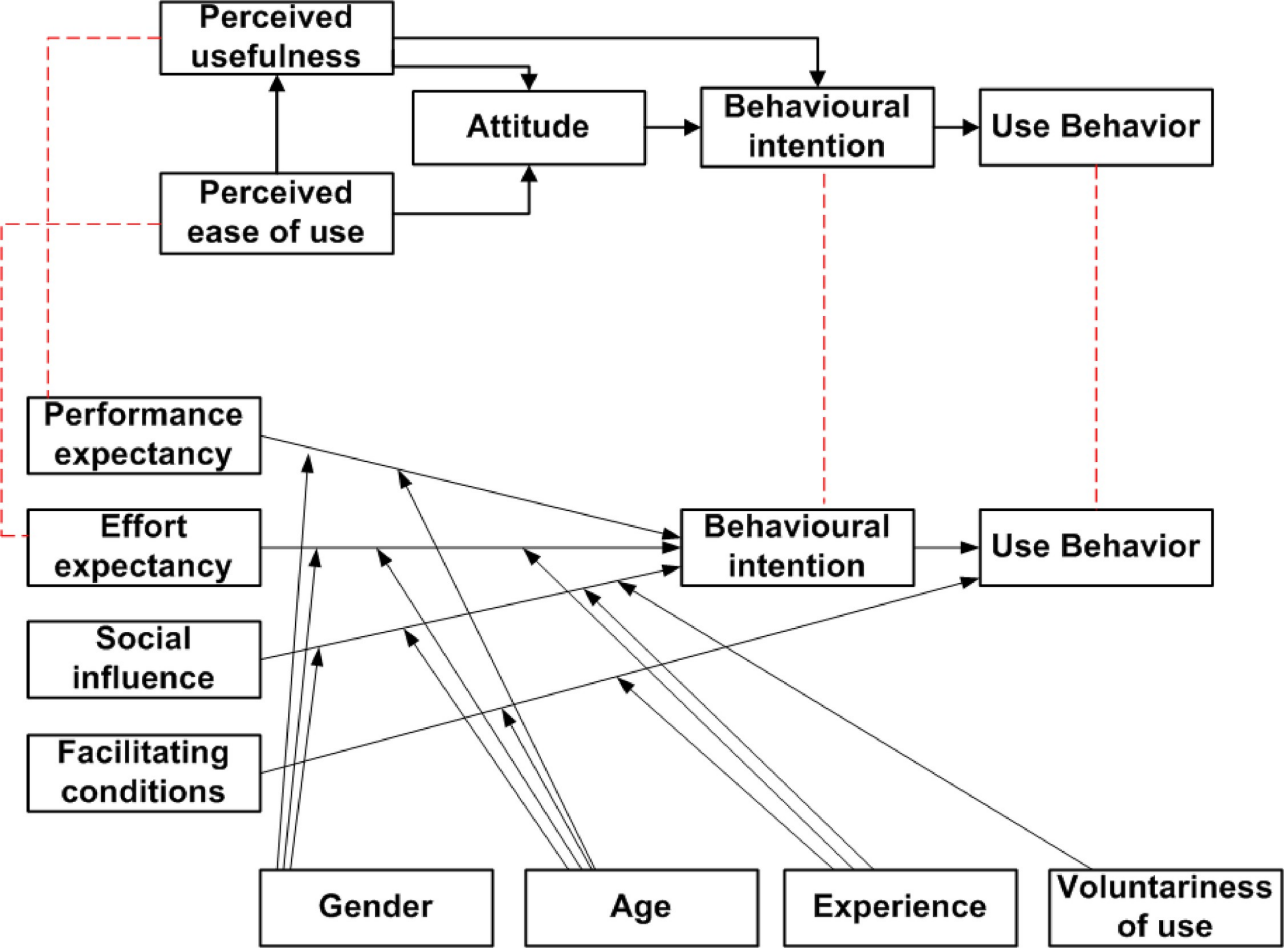
Comparison between the TAM and UTAUT models [47].

Besides, a comparison between TAM and UTAUT shows that perceived usefulness in TAM corresponds to performance expectancy in the UTAUT model while perceived ease of use in TAM corresponds to effort expectancy. Also, behavioural intention and actual usage are provided by both TAM and UTAUT models [14, 47]. However, the UTAUT model does not include the attitude construct in TAM as illustrated in Fig 3.

Prior studies have extended the UTAUT model contextually. For instance, Pal et al. [18] explored the main factors influencing the acceptance of smart home services for healthcare by the elderly. The authors used the original four core factors of the UTAUT model and extended it with another four contextual factors namely technology anxiety, perceived trust, perceived cost, and expert advice.

Another study [24] examined the adoption of smart home services among 239 elderly people by testing three theoretical models: the TAM, TRA and TPB. This study showed that the selected three models are valid but lack some factors. Ashraf et al. [48] focused on understanding the perception and post-adoption behavioural of old people towards SHTs in Pakistan. Authors in [49] focused on the factors that impact the residents’ acceptance of smart homes. The authors extended the TAM model with other factors and found that perceived usefulness, perceived ease of use, trust, level of awareness, enjoyment, and perceived risks; are the main predictors of residents’ attitudes regarding smart homes. Gross et al. [3] identified the main factors that affect the consumer adoption of SHTs among consumers in Germany. This study extended the TAM using comfort, security, and cost features. However, contextual factors relating to Saudi Arabia are yet to be examined. As such, examining the contextual factors would further extend UTAUT and enhance its predictability.

## 3. Research framework and hypotheses

### 3.1 Underlying theory–UTAUT model

The UTAUT theory is one of the theories in technology literature to explain technology awareness, usage, and acceptance. Several reasons account for the use of the UTAUT model in explaining the variations in behavioural intention to use SHT. First, the UTAUT theory has been well-validated in technology acceptance research as an accurate predictor of system usage and acceptance [7]. Thus, it suggests that the UTAUT theory provides an avenue to test whether the users of technology accept or reject it. Second, in developing the UTAUT theory, Venkatesh et al., (2003) combined eight specific models and theories including TRA, TPB, and TAM to document the determinants of behavioural intention and usage behaviour of technology. Third, unlike past theories predicting behaviour towards technology usage, UTAUT theory takes into consideration demographic factors such as age and gender. Importantly, [7] suggests that future studies should enhance the UTAUT theory by further testing contextual factors since the UTAUT theory was developed based on the underlying. Therefore, this study advances the UTAUT theory with the inclusion of contextual and human behaviour factors (Culture influence, technology awareness, attitude, education, region) within an Arab setting. Originally, four factors of the UTAUT model are direct predictors (performance expectancy, effort expectancy, social influence, and facilitating conditions) of behavioural intention [50] as discussed below.

### 3.2 Hypotheses development

**Fig 4:**
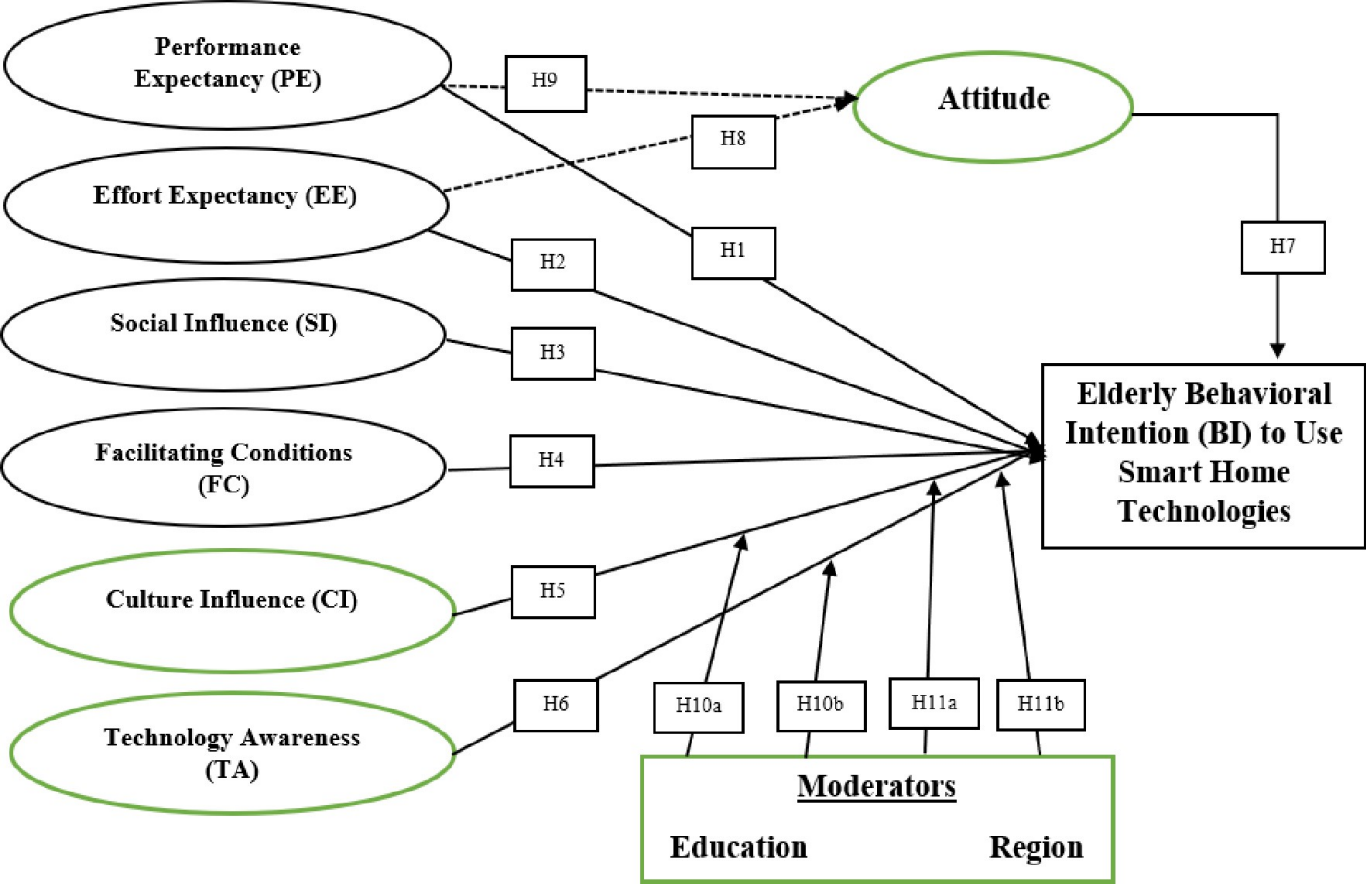
Proposed research framework.

This study formulates some hypotheses on the main UTAUT variables (performance expectancy, effort expectancy, social influence, and facilitating conditions), external/contextual variables (culture influence and technology awareness), moderating variables (education and region), and mediating variables (attitude). Hypotheses were formulated on these variables and linked to behavioural intention to use smart home technologies. The theoretical framework connecting these variables and relationships is shown in Fig 4.

#### 3.2.1 Performance expectancy (PE)

Venkatesh et al. [7] define performance expectancy as" the degree to which using technology will provide benefits in performing certain activities". SHTs are beneficial for the elderly and they often increase their users’ intention to use new technology. However, fear and uncertainty about the usability of new technology can lead to a negative perception and decrease its adoption.

A higher expectation of benefits from Smart Home Technologies SHT by elderly people, such as perceived better management of their daily activities, better access to health services, and a higher quality of life in general, will have a positive impact on older users’ intention to use SHT [41, 51]. By accepting the original definition of PE to the current context, we have defined this factor as the extent to which individuals believe that using the IoT will help them accomplish a particular task.

**H1:** Performance expectancy positively impacts the BI to use smart home technologies among the elderly.

#### 3.2.2 Effort expectancy

Effort expectancy is defined as [7] “the degree of ease associated with the use of any system". The effort expectancy has a strong effect on the elderly intention toward smart home technology and an important positive effect on their acceptance of new technology. Thus, the easiness to learn and using technology increases its usefulness. Consequently, a higher expectation of effort refers to the energy required to manage the system. In the context of end-user using connected smart home devices, effort expectancy is also associated with a higher perception of the technologies, as being more beneficial and useful [52]. The ease of use of technology will affect the user’s perception of the technology, making effort expectancy the antecedent of behavioural intention [43].

**H2:** Effort expectancy positively impacts the BI to use smart home technologies among the elderly.

#### 3.2.3 Social influence

The degree to which an individual’s use of new technology is influenced by the people in his or her social group is described as a social influence [53]. Prior studies have established that social influence has a positive link with behavioural intention to use a new technology [12, 54]. In contrast, [55] found that social influence has an insignificant impact on behavioural intention to use, due to reduced social interactions. This suggests that when social groups are devoid of interactions, influencing partners and group members to use technology becomes difficult. For instance, the Covid-19 pandemic resulted in many state lockdowns and movement restrictions, which affects the social influence of online technology systems since social interactions are reduced. Similarly, [56] noted that ageism can also contribute to elderly people’s social exclusion, thus, limiting the social integration required to influence technology acceptance and usage. Meanwhile, concerning smart home technologies for elderly people, with the sole purpose to monitor the health status of adults, older adults often perceive social activity and connectedness to be strongly associated with their mental health. This suggests that the elderly’s influence to use technology is linked to their mental health. [57] supported that social connectedness among elderly people influences their social and mental health since adults who are associated with loneliness exhibit poorer overall health. This supports the notion that an individual adult’s usage of smart home technology may further influence another elder’s perception of the technology with a social integration system. For such new technology, a user’s inadequate knowledge of the technology may be enhanced by the opinions of his/her social members, friends, and family Hence, there is a positive relationship between the social influence and the usage intention [24].

**H3:** Social influence positively impacts the BI to use smart home technologies among the elderly.

#### 3.2.4 Facilitating conditions

Venkatesh et al. [7] defined facilitating conditions as “the degree to which an individual believes that an organizational and technical infrastructure exists to support the use of the system". It directly affects the BI to use a new technology [43]. However, the relationship between facilitating conditions and BI to use is dependent on age. Studies documenting a positive relationship between facilitating conditions and BI to use SHTs have surveyed people without no reference to a specific group (e.g., young, old, generations x, y, z). Meanwhile, prior studies that found negative or insignificant relationships by surveying elderly people argued that older adults tend to rely less on evolving technology due to their unique characteristics [43, 58, 59]. [43, 47] further stressed that elderly people often perceive smart home technologies as immature, and as such may be reluctant to use the technology. Thus, studies focusing on elderly people are more likely to find the negative or insignificant impact of facilitating conditions on BI to use SHTs. [60] attributed such insignificance of facilitating conditions on BI to use SHTs to a lack of equipped knowledge and skills to use smart home technologies by elderly people. Therefore, since the present study focuses on elderly people, we conjecture that:

**H4:** Facilitating conditions negatively impact the BI to use smart home technologies among the elderly.

#### 3.2.5 Culture influence

Elderly people tend to think and reason in the way they learn from living in a special technology environment [61]. Older adults are influenced to use technologies to complete their work-related tasks due to convenience and the low-cost-related attributes of the new technology. Elderly people’s cultural belief is that they have better access to information especially as it relates to their health problems. A few studies have been conducted on the link between culture influence and behavioural perception of elderly people [62]. However, similar studies are absent in Saudi Arabia where social activities are determined by a strong cultural belief system. This is in line with the call for more investigation on culture influence and behavioural perception in the Arab content [10]. In addition, [12] have documented that cultures and social-psychological factors are among the barriers to the successful adoption of new technology, contending that different local values have differences in behavioural intention. In Saudi Arabia, the privacy of new technology is often considered as a cultural value since Islamic injunctions specify the acceptance of the technology in their ways of life. Thus, as suggested by [12] that cultural settings across different regions may play an impact on behavioural intention, we hypothesize that:

**H5:** Culture influence has a positive relationship with BI to use smart home technologies among the elderly.

#### 3.2.6 Technology awareness

Awareness is defined by Shuhaiber and Mashal as “an understanding that allows the user to reduce uncertainty in a subjective manner" [49]. It has a potential impact on the adoption of smart technologies. Usually, the elderly decides whether to accept or reject a new technology after having complete knowledge of it. Elderly people should know about smart technologies and how to use them. Coughlan et al. [63] proved that technology awareness is a relevant predictor of SHTs adoption. Hence, companies should take more efforts to increase the level of SHT adoption among elderly people [33, 64]. Technology awareness reduces the technological reluctance in people, in a way to increase their intention to use a new technology [65] As such, elderly people who have the basic knowledge about the benefits of SHTs and guidance on how these smart technologies work are less reluctant to adopt SHTs [66]. Therefore, it is hypothesized that:

**H6:** Technology awareness positively impacts the BI to use smart home technologies among the elderly.

#### 3.2.7 Attitude

Attitude represents a mediator factor in TAM [7] and has been documented as a major predictor in technology acceptance models, which partially mediates the effects of independent constructs on BI [67]. Thus, individuals with a positive attitude towards smart homes are more likely to accept SHTs than those with a negative attitude. This present study also examines the effect of attitude and BI to use SHTs among elderly people in Saudi Arabia.

According to [9], attitude plays a significant influence on behaviour and intention to use. Thus, many empirical studies have examined the relationship between attitude and behaviour intention to use technology and found a positive relationship [10, 42, 68]. This suggests that individuals with an overall positive attitude toward behavioural intention to use SHTs are more likely to accept SHTs than those with a negative attitude toward the technologies. Some studies have found that the explanatory power of BI increases when the attitude is included in the UTAUT model [69, 70]. This present study aims to investigate the effect of attitude and BI to use SHTs among elderly people in Saudi Arabia.

**H7:** Attitude has a positive impact on BI to use smart home technologies among the elderly.

#### 3.2.8 Mediating role of attitude on the relationships between EE, PE, and BI to use SHTs

Meanwhile, as indicated in the TAM theory, attitude plays a mediating role between PEOU and intention. However, no studies have investigated how attitude performs the role of a mediator on the link between the independent variables and behavioural intention in the UTAUT model to measure elderly behavioural intention to use SHT. Supporting this claim, [71] argues that a mediator is necessary when studies are confirming no direct relationship between two constructs. A few empirical studies on behavioural intention have documented no direct relationship between effort expectancy, performance expectancy and behavioural intention, thus, justifying the need for a moderator [70]. Therefore, this present study introduces attitude as a mediator between effort expectancy, performance expectancy, and elderly behavioural intention to use SHTs. Therefore, we formulate that:

**H8:** Attitude positively mediates the relationship between effort expectancy and BI to use smart home technologies among the elderly.
**H9:** Attitude positively mediates the relationship between performance expectancy and BI to use smart home technologies among the elderly.

#### 3.2.9 Moderating the role of education on the relationships between culture influence, technology awareness and BI to use SHTs

A moderating variable is an interactive term that appears when the association between independent and dependent variables is surprisingly weak, inconsistent, or non-existent. It is used to lessen or strengthen the relationship [72, 73]. Based on this, this study uses education to moderate the relationship between culture, technology awareness and elderly behavioural intention to use SHTs, following the research gap in technology acceptance literature on moderators. This is to [10, 11, 74] to strengthen the relationship between culture, technology awareness, and BI. Thus, this present study introduced education as a moderating variable on the relationship with culture followed by technology awareness on elderly BI to use SHTs. The following hypotheses were formulated:

**H10a:** The influence of culture on elderly BI is moderated by education.
**H10b:** The influence of technology awareness on elderly BI is moderated by education.

#### 3.2.10 Moderating role of region on the relationships between culture influence, technology awareness and BI to use SHTs

There is practical evidence in Saudi Arabia that the Western region, the Central Region, and the Eastern Region are more technologically advanced. Oftentimes, new technologies are adopted early in these regions while other regions like the Southern and Northern regions are known for late adoption behaviour. Thus, the level of technology awareness in the Western region, the Central Region, and the Eastern Region seems to be high when compared to other regions. Thus, regional development may be responsible for the level of technology awareness which would further influence the behavioural intention to use SHTs. It is expected that the level of technology awareness in regions that are more commercialized, or business dominated would enhance the variations in BI to use SHTs. Elderly people in these regions are more likely to be aware of new technologies in the market and how such technologies such as SHTs could impact their lives positively.

The Arab society is generally characterized as being religious, family-oriented, and traditionally conservative. In Saudi, the people’s attitudes and traditions follow those of the old centuries, derived from Arab civilization and Islamic heritage. However, findings have shown a positive relationship between culture influence and BI [10, 75]. But no studies have examined the moderating role of the region as a significant factor to determine the strength of that relationship. Therefore, this research examines the moderating role of the region on the relationship between cultural influence and BI, followed by technology awareness on elderly behavioural intention to use SHTs. Therefore, the hypothesis in that regard was formulated as follows:

**H11a:** The influence of culture on BI is moderated by region.
**H11b:** The influence of technology awareness on BI is moderated by region.

## 4. Methodology

This section introduces the validity and reliability of the study instrument. In addition, it provides the details of the data collection and analysis.

### 4.1 Survey instrument

This study uses the survey approach by using questionnaires to elicit responses from respondents on the variables examined. The instrument has three sections. First, it presents the demographic details of the respondents such as gender, age group, region, education level, living status, and monthly income group.

The second section of the instrument has various statements that are used to measure the constructs of the study. The statements on the constructs in the proposed research framework used in this study have been adopted from previous studies and adapted in the study to suit the Saudi context. The third part of the instruments asked a few open-ended questions on other factors that could impact the intention of the elderly to use SHTs. Moreover, the study adopts a 5-point Likert scaling starting from 1-strongly disagree to 5- strongly agree to enable the numerical analysis of the relationships between the variables studied.

### 4.2 Validity and reliability

The study adopts expert validity to validate the items used in measuring the constructs in the research instrument [76, 77]. We confirm the reliability of the questionnaire by surveying 87 respondents during the pilot study. The results (using Cronbach alpha) confirm that the instrument is valid and reliable. Thus, there is no issue with the instruments and the data collected are valid for analyses of the research hypotheses [78]. The questionnaire was administered via an online survey tool called Google Forms [50].

### 4.3 Sampling design

Sampling is a procedure of selecting a sample from a population as a representation of the behaviours of the whole population [72]. This study uses the purposive sampling technique in selecting the respondents of the study. [72] noted that the investigator uses the purposive sampling technique when there are exclusion criteria or when the investigator believes that a category of participants is fit to take part in this study. Prior studies in technology awareness and elderly people have adopted the purposive sampling technique [17].

### 4.4 Common method bias (CMB)

Podsakoff et al. [79] noted that survey research cannot be free of sampling bias. Thus, it suggests that the issue of common method bias should be addressed in behavioural research. Although several studies have used Harman’s single factor to address the issue of CMB [43, 80], there has been considerable evidence that the single factor loading testing is not strong enough. In the light of this, [81] suggested the use of full collinearity using the variance inflation factor with a value, not more than the 3.33 threshold. We address any possible CMB in our study by performing the VIF test and the results are presented in Table 1. The VIFs values are well below the 3.33 threshold value suggested by [81]. Thus, the study confirms that there is no CMB problem in the survey data.

**Table 1:** Full collinearity.

Construct	Full collinearity (VIF)	
	Attitude	Behavioural intention
Performance expectancy	1.025	1.763
Effort expectancy	1.025	1.398
Social influence		1.308
Facilitating condition		1.202
Culture influence		2.340
Technology awareness		2.455
Education		1.268
Region		1.289
Attitude		2.482
Behavioural intention		-

### 4.5 Data collection and analysis

The questionnaire was used to collect data through various online platforms (Email, LinkedIn, and WhatsApp). The questionnaire link was shared on online platforms with more than 1,000 group members and families in Saudi Arabia to target the elderly. As designed, incomplete questionnaires are not permitted to be submitted since all the constructs’ items are mandated to be completed. Through this approach, a total of 486 completed questionnaires were collected. The sample size of 486 is quite higher than the recommended size of 100–150 for a model that contains seven or fewer constructs as suggested by [82].

The sample size also confirms the recommendation of Hair et al. which suggests ten times the latent variable with the highest number of predictors [83]. Furthermore, Creswell [84] recommended a sample size in the range of 350 and 500 for good quantitative research. Therefore, the sample size of 486 is adequate to analyse the constructs of the study’s model.

As illustrated in Table 2, The results depict that about 38.68% of the participants were males (n = 188), while the rest were females (n = 298). The age group shows that about 56.38% represents elderly people aged between 60 and 69 years, 24.28% are in the age group 70–79 years, and 19.34% for those who are 80 years old and above. Concerning nationality, the results depict that about 90.12% are Saudi citizens while the rest 9.88% are non-Saudis. The results for the region reveal that about 31.48% of the participants are from the Western region (N = 153), about 26.54% (N = 129) of the elderly are from the Central Region, and about 22.43% of the respondents are residing in the Eastern Region, and about 13.17% stay in the Southern region, and 6.38% in the Northern region. In addition, the study uses the Smart PLS 3.2.8 software to analyse the data collected from respondents [47]. Smart PLS is appropriate to analyze multiple and complex relationships at a time by avoiding undesirable problems and avoiding the pitfalls of first-generation software [85]. As required, data collected from respondents were saved in Microsoft Excel using the Comma Separated Values file (.csv) before uploading to the Smart PLS for analysis to allow the software reads the data and enables the analysis of measurement and structural model assessments.

**Table 2:** Descriptive statistics of demographic characteristics of participants.

Demographics	Category	No. of Participants	Frequency
Age	60–69 years	274	56.38%
	70–79 years	118	24.28%
	80 years & above	94	19.34%
Gender	Male	188	38.68%
	Female	298	61.32%
Nationality	Saudi	438	90.12%
	Non-Saudi	48	9.88%
Region	Central Region	129	26.54%
	Western Region	153	31.48%
	Southern Region	64	13.17%
	Eastern Region	109	22.43%
	Northern Region	31	6.38%
Education	Illiterate	76	15.64%
	Lower secondary	110	22.63%
	Upper secondary	141	29.01%
	Degree	132	27.16%
	Master	20	4.12%
	Ph.D.	7	1.44%
Living status	Living alone	40	8.23%
	Living with relatives	75	15.43%
	Living with family members	356	73.25%
	Others	15	3.09%
Income	Less than R1500	189	38.89%
	SR1500-SR3000	126	25.93%
	SR3000-SR5000	58	11.93%
	SR5000-SR10000	113	23.25%

## 5. Results and discussion

This section is divided into two sub-sections: 5.1 and 5.2. The results were provided in section 5.1 while section 5.2 provides the discussion of the results.

### 5.1 Results

We provide the results for the measurement and structural models in this subsection. The essence is to confirm the reliability and validity of our constructs and items. The structural model assessment is to confirm the formulated hypotheses.

#### 5.1.1 Results for the measurement model

This paper examines the elderly behavioural intention toward accepting the use of use smart home technologies in Saudi Arabia. Hair et al. [83] suggested that the measurement model is assessed by performing (i) convergent validity and reliability, and (ii) discriminant validity using the HTMT criterion [85].

*Convergent validity and reliability*. The results provided in Table 3 showed that all the constructs have average variance extracted (AVE), composite Reliability(CR), and Cronbach’s alpha (CA) scores greater than their threshold scores of 0.5, 0.7, and 0.7, respectively [86]. The AVEs scores are in the range of 0.521 and 0.791, greater than the 0.50 recommended [85–87].

**Table 3:** Convergent validity.

Constructs	Items	Loadings	VIF	AVE	CR	CA
Performance Expectancy	PE1	0.854	2.191	0.674	0.912	0.880
	PE2	0.815	1.985			
	PE3	0.801	2.142			
	PE4	0.817	2.206			
	PE5	0.817	2.020			
Effort Expectancy	EE1	0.645	1.109	0.547	0.825	0.716
	EE2	0.608	1.241			
	EE3	0.831	2.447			
	EE4	0.843	2.509			
Social Influence	SI1	0.815	2.375	0.737	0.933	0.911
	SI2	0.882	3.084			
	SI3	0.870	2.621			
	SI4	0.913	3.660			
	SI5	0.806	2.088			
Facilitating Condition	FC1	0.922	5.025	0.748	0.936	0.938
	FC2	0.940	4.894			
	FC3	0.767	3.303			
	FC4	0.797	4.634			
	FC5	0.884	4.698			
Culture Influence	CI1	0.857	2.609	0.774	0.945	0.927
	CI2	0.887	3.055			
	CI3	0.892	3.121			
	CI4	0.902	3.422			
	CI5	0.860	2.589			
Technology Awareness	TA1	0.800	1.851	0.600	0.881	0.829
	TA2	0.852	2.277			
	TA3	0.771	1.692			
	TA4	0.828	1.995			
	TA5	0.594	1.254			
Education	Education	1.000	1.000	1.000	1.000	1.000
Region	Region	1.000	1.000	1.000	1.000	1.000
Attitude	ATT1	0.619	1.307	0.521	0.844	0.770
	ATT2	0.711	1.511			
	ATT3	0.732	1.507			
	ATT4	0.770	1.615			
	ATT5	0.767	1.561			
Behavioural Intention	BI1	0.887	2.748	0.791	0.938	0.912
	BI2	0.895	2.972			
	BI3	0.906	3.229			
	BI4	0.868	2.460			

The constructs have composite reliability scores between 0.825 and 0.948, greater than the 0.70 suggested by Chin et al. [85, 87, 88]. Also, the CA scores are in the range of 0.716 to 0.938, acceptable and higher than the 0.70 suggested [89]. In addition, the VIF values range from 1.109 to 2.741, quite lower that the 3.33 threshold score recommended by Petter et al. [90]. Thus, indicating that there is no multicollinearity problem in the explanatory variables [91].

Table 3 also provides the results for the item loadings, with all items having loadings between 0.50 and 0.60, which are far higher than the thresholds recommended by [89, 92].

*Discriminant validity*. Table 4 provides the results for the cross-loadings as depicted in the diagonal values (with green highlights). According to Hair et al. [83], every item of a construct must have a greater loading on its construct than any other items. The results revealed that the cross-loadings show adequate discriminant validity, which also satisfies the suggestion of Gefen et al. [93, 94] that a reflective item should load greater on their constructs than others to assess the discriminant validity [93, 94].

**Table 4:** Cross-loadings.

Constructs	Items	1	2	3	4	5	6	7	8	9	10
1. Attitude	ATT1	0.619	0.306	0.250	0.367	0.006	-0.043	0.178	0.067	0.055	0.360
	ATT2	0.711	0.380	0.289	0.435	-0.060	0.000	0.187	0.048	0.305	0.591
	ATT3	0.732	0.411	0.328	0.402	-0.027	-0.033	0.202	0.013	0.283	0.588
	ATT4	0.770	0.480	0.513	0.308	0.041	-0.032	0.392	-0.019	0.302	0.549
	ATT5	0.767	0.530	0.544	0.337	0.031	0.014	0.440	0.013	0.305	0.501
2. Behavioural Intention	BI1	0.501	0.887	0.638	0.424	0.007	-0.073	0.525	0.039	0.279	0.470
	BI2	0.503	0.895	0.664	0.342	-0.024	-0.093	0.568	0.023	0.306	0.527
	BI3	0.570	0.906	0.621	0.362	0.036	-0.037	0.506	0.019	0.359	0.558
	BI4	0.541	0.868	0.636	0.342	0.046	-0.052	0.501	0.003	0.386	0.533
3. Culture Influence	CI1	0.466	0.591	0.857	0.276	0.043	-0.048	0.546	-0.009	0.330	0.493
	CI2	0.502	0.630	0.887	0.299	0.041	-0.067	0.554	0.003	0.339	0.489
	CI3	0.466	0.662	0.892	0.253	0.084	-0.029	0.577	-0.052	0.324	0.510
	CI4	0.484	0.641	0.902	0.265	0.046	0.035	0.565	-0.017	0.383	0.478
	CI5	0.504	0.636	0.860	0.240	0.037	0.021	0.524	-0.019	0.390	0.494
4. Effort Expectancy	EE1	0.421	0.358	0.280	0.645	-0.108	-0.073	0.148	0.063	0.294	0.452
	EE2	0.292	0.201	0.141	0.608	0.019	-0.013	0.116	0.067	0.145	0.258
	EE3	0.359	0.274	0.172	0.831	0.026	0.004	0.054	0.000	0.008	0.303
	EE4	0.385	0.345	0.263	0.843	0.032	0.006	0.130	0.026	0.043	0.290
5. Education	Education	0.001	0.018	0.058	-0.018	1.000	0.316	-0.038	-0.401	0.011	-0.001
6. Facilitating Condition	FC1	0.010	-0.052	-0.004	0.000	0.325	0.922	-0.085	-0.290	0.058	0.030
	FC2	-0.009	-0.061	-0.012	-0.034	0.355	0.940	-0.073	-0.320	0.070	-0.004
	FC3	0.038	0.022	0.049	0.000	0.293	0.767	-0.024	-0.286	0.059	0.064
	FC4	-0.021	-0.001	0.024	-0.010	0.229	0.797	-0.056	-0.255	0.098	0.018
	FC5	-0.041	-0.054	-0.013	-0.034	0.203	0.884	-0.066	-0.274	0.060	0.013
7. Performance Expectancy	PE1	0.407	0.565	0.659	0.128	-0.022	-0.049	0.854	0.035	0.321	0.361
	PE2	0.328	0.527	0.536	0.179	-0.025	-0.063	0.815	0.086	0.246	0.288
	PE3	0.322	0.384	0.359	0.043	-0.039	-0.105	0.801	0.142	0.117	0.192
	PE4	0.248	0.407	0.473	0.084	-0.025	-0.108	0.817	0.082	0.146	0.197
	PE5	0.327	0.499	0.502	0.179	-0.049	-0.051	0.817	0.131	0.237	0.277
8. Region	Region	0.029	0.023	-0.022	0.052	-0.401	-0.317	0.112	1.000	-0.076	-0.007
9. Social Influence	SI1	0.233	0.219	0.259	0.131	0.003	0.068	0.131	-0.057	0.815	0.266
	SI2	0.342	0.307	0.347	0.218	0.021	0.064	0.216	-0.056	0.882	0.377
	SI3	0.382	0.385	0.448	0.144	-0.008	0.048	0.295	-0.082	0.870	0.425
	SI4	0.315	0.344	0.367	0.132	0.021	0.065	0.266	-0.082	0.913	0.383
	SI5	0.229	0.308	0.254	0.121	0.009	0.054	0.213	-0.040	0.806	0.295
10. Technology Awareness	TA1	0.571	0.458	0.503	0.299	-0.007	-0.009	0.285	-0.043	0.281	0.800
	TA2	0.624	0.479	0.464	0.356	-0.017	0.006	0.302	-0.017	0.350	0.852
	TA3	0.578	0.461	0.449	0.444	0.026	0.063	0.262	0.048	0.294	0.771
	TA4	0.560	0.509	0.429	0.372	-0.027	0.032	0.231	-0.026	0.398	0.828
	TA5	0.439	0.350	0.309	0.279	0.032	-0.104	0.194	0.017	0.276	0.594

The study assesses the discriminant validity using the HTMT criterion. According to Hair et al. [83], all the values in the HTMT must be less than 0.90. As presented in Table 5, the results show that all the values are between 0.024 and 0.898, which are less than 0.90, suggesting that the constructs fulfil the discriminant validity.

**Table 5:** Discriminant validity (HTMT criterion).

Constructs	1	2	3	4	5	6	7	8	9	10
1. Attitude	-									
2. Behavioural Intention	0.697									
3. Culture Influence	0.631	0.782								
4. Education	0.052	0.033	0.059							
5. Effort Expectancy	0.679	0.496	0.358	0.075						
6. Facilitating Condition	0.049	0.054	0.050	0.325	0.047					
7. Performance Expectancy	0.462	0.647	0.682	0.041	0.192	0.083				
8. Region	0.051	0.025	0.024	0.401	0.063	0.329	0.124			
9. Social Influence	0.403	0.400	0.425	0.015	0.210	0.084	0.279	0.077		
10. Technology Awareness	0.898	0.674	0.638	0.031	0.576	0.082	0.376	0.043	0.468	-

#### 5.1.2 Results for the structural model

**Fig 5:**
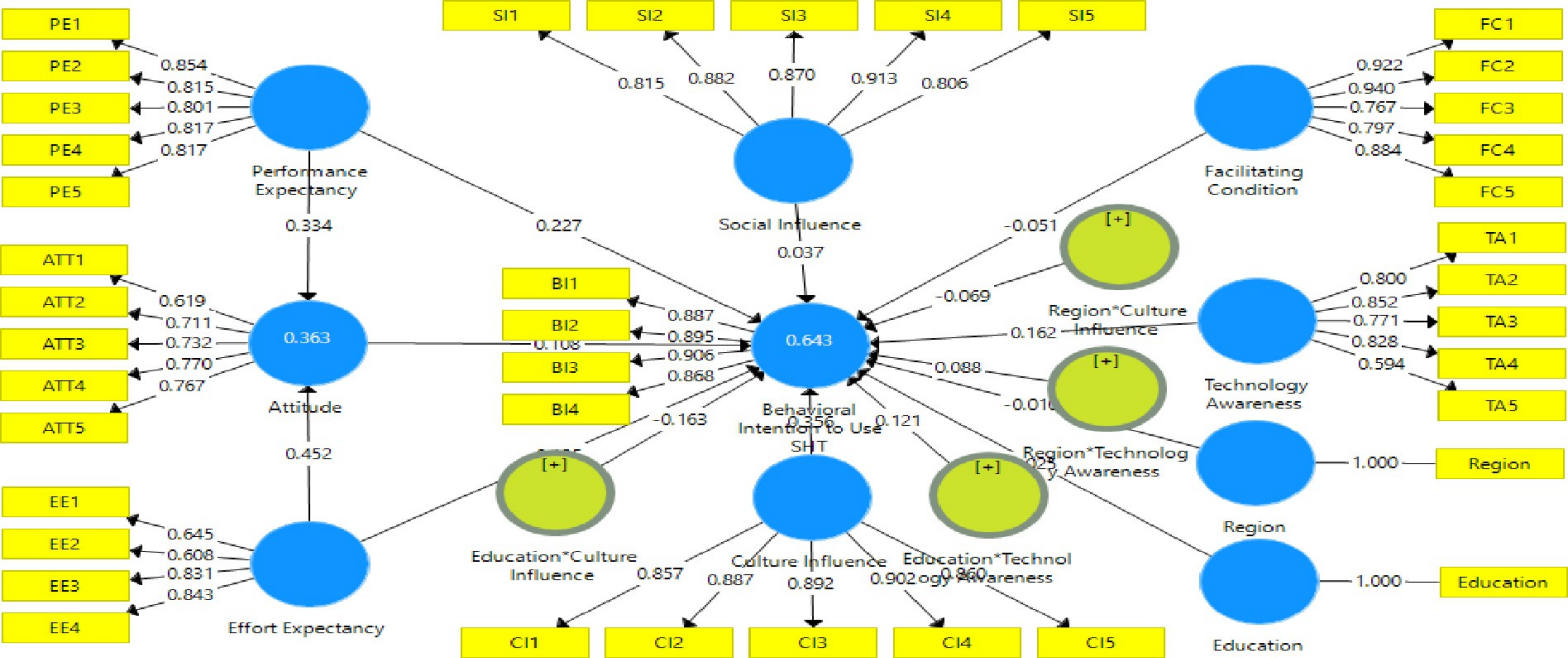
Structural model assessment.

This section provides the results for the structural model after confirming the results of the measurement model. Fig 5 shows the graphical respresentation of the results for the structural model. The R-square values are shown in the dependent variables’ ovals. PE and EE explain about 36.30% variations in attitude while all the explanatory variables explain about 64.30% variations in behavioral intention to use SHT. The essence is to test the formulated hypotheses as indicated in the proposed research framework. The results for the structural model are depicted in Table 6.

**Table 6:** The results of the structural model.

Direct Hypotheses	Beta	Std.	T-value	P-Values	R²	Decision
***H1*:** Performance Expectancy -> Behavioural Intention	0.227	0.040	5.705	0.000	0.643	Supported***
***H2*:** Effort Expectancy -> Behavioural Intention	0.135	0.033	4.085	0.000		Supported***
***H3*:** Social Influence -> Behavioural Intention	0.037	0.033	1.138	0.128		Not Supported
***H4*:** Facilitating Condition -> Behavioural Intention	-0.051	0.056	0.915	0.180		Not Supported
***H5*:** Culture Influence -> Behavioural Intention	0.356	0.050	7.063	0.000		Supported***
***H6*:** Technology Awareness -> Behavioural Intention	0.162	0.043	3.796	0.000		Supported***
***H7*:** Attitude -> Behavioural Intention	0.108	0.043	2.478	0.007		Supported***
**Indirect/Mediating Hypotheses**						
***H8*:** Effort Expectancy -> Attitude -> Behavioural Intention	0.049	0.020	2.403	0.008		Supported***
***H9*:** Performance Expectancy -> Attitude -> Behavioural Intention	0.036	0.015	2.413	0.008		Supported***
**Moderating Hypotheses**						
***H10a*:** Education*Culture Influence -> Behavioural Intention	-0.163	0.045	3.637	0.000		Supported***
***H10b*:** Education*Technology Awareness -> Behavioural Intention	0.121	0.043	2.787	0.003		Supported***
***H11a*:** Region*Culture Influence -> Behavioural Intention	-0.069	0.045	1.521	0.064		Not Supported
***H11b*:** Region*Technology Awareness -> Behavioural Intention	0.088	0.048	1.830	0.034		Supported**

The structural model is evaluated using the R2 of the endogenous constructs. As recommended by Chin [95], R-squared values above 0.67 are considered adequately high, a range of 0.33–0.67 is considered to be moderately adequate while values ranging from 0.19 to 0.33 are considered weak. Meanwhile, R2 values less than 0.19 are unacceptable [96]. As depicted in Table 6, the result shows an R2 of 0.643, suggesting that about 60.42 per cent of the variances in BI is explained by all the exogenous variables in the model.

### 5.2 Robustness test

We further re-examine the structural model of study by excluding education. Although the region shows mixed findings, however, the interpretations are in line with contextual practices in Saudi Arabia. However, the moderating role of education on culture influence seems to underline the general belief that education would decline culture influence on behavioural intention to use SHTs.

To compare the alternative model with the focal model on the suitability to exclude education as a moderator, as depicted in Table 7, the study finds that the focal model is better than the alternative model given the values of AIC and BIC. The values of AIC and BIC of the focal model are higher than that of the alternative model as supported by previous studies [97, 98]. In addition, as suggested by [83] that the EN score should be higher than 0.50, the focal model has an EN score higher than the EN value of the alternative model. Thus, the focal model is better and has better goodness of fit.

**Table 7:** Results of BIC, AIC and EN.

The goodness of fit indicators	The study’s focal model	The alternative model
AIC (Akaike’s Information Criterion)	1852.243	1873.138
BIC (Bayesian Information Criteria)	1998.760	2002.910
EN (Entropy Statistic (Normed))	0.833	0.800

### 5.3 Discussions

This subsection discusses the findings with previous studies and contextual evidence. It also discusses the findings concerning the UTAUT theory.

#### 5.3.1 Findings of the direct hypotheses

This section explains the direct relationships between the variables. The result of the findings from data analysis in this research was discussed. The discussion will begin with the direct relationship between performance expectancy and BI, followed by effort expectancy, social influence, facilitating condition, culture influence, and technology awareness on behavioural intention. These analyses will pursue the mediating effect of attitude between performance expectancy as well as the moderating effect of education and region between culture influence and technology awareness.

*Relationship between performance expectancy and BI*. The finding for H1 indicated that performance expectancy has a positive and significant effect on the elderly’s BI to use SHTs. Interestingly, the finding is quite aligned with some of the researchers Sidorova [67]; Almetere [71] and Mashal and Shuhaiber [2] who have proposed that the performance expectancy has a linear relation with behavioural intention. Also, different studies have established the fact that performance expectancy affects behavioural intention in various technology applications. For instance, [99] highlighted that those with higher performance expectancy lead to higher behavioural intention to use enterprise resource planning software. [99] discovered that performance expectancy and behavioural intention to accept information technology signal a significant and positive relationship.

Venkatesh et al. [7] discovered that performance expectancy significantly and positively influences one’s BI to accept and use an IT system. However, this present study contributes to the past findings by making it clear through its finding that performance expectancy has a positive and significant effect on BI to use SHTs among older people in Saudi Arabia.

*Relationship between effort expectancy and BI*. The second hypothesis (H2) of the study is whether effort expectancy has a positive effect on BI to use SHTs. The research found that effort expectancy has a positive effect on behavioural intention to use SHTs. The results are in line with the past studies [67, 71] which also found that effort expectancy has a positive effect on BI. Additionally, other studies also found similar results from different technology applications. [100] the finding revealed that effort expectancy is a significant determinant of the adoption of mobile learning. This present study was able to establish the fact that effort expectancy positively and significantly influences BI to use SHTs among the elderly in Saudi Arabia because when these people feel that SHTs are easy to be used to foster or improve their needs, their behavioural intention to use SHTs will increase.

*Relationship between social influence and BI*. The third hypothesis of the study (H3) examines whether social influence has a significant and positive effect on BI to use SHTs among the elderly. But the findings show contrary to other findings that social influence has no significant on BI to use SHTs among elderly people in Saudi Arabia. The result of this finding is supported by [101] who also found no relationship between social influence and BI. However, because the social impact was not significant, some studies had to reject the hypothesis [102]. However, [103] found that social influence is positively significant to older adults’ intention to use the information and communications technology (ICT) and [66] also found in their study that social influence is positively correlated with BI.

The present study is unique in the sense that it focused basically on older people in the kingdom of Saudi Arabia. This result proves that these older people are influenced when people around them such as friends, family, and colleagues are using SHTs. Due to this subjective norm, the users will try to engage with them and derive an intention to use SHTs.

*Relationship between facilitating condition and BI*. The fourth hypothesis of the study (H4) examines whether facilitating condition has a significant and positive effect on BI to use SHTs among the elderly. Just like every other component of UTAUT. The term facilitating conditions is also one of the basic constructs of the UTAUT Model by [7]. The findings show that facilitating conditions have no significant effect on BI to use SHTs among elderly people in Saudi Arabia. This is supported by [24, 104] who also found a negative effect of facilitating conditions on behavioural intention.

The elderly in Saudi Arabia feel that the needed facilitating conditions such as required resources and adequate facilities are not perceived. Therefore, it is difficult for less-experienced users to use SHTs for their welfare while experienced users tend to be the enabler to independently acquire resources or support.

*Relationship between culture influence and BI*. The fifth hypothesis (H5) of the study is to examine whether culture influence is positively related to behavioural intention. Culture plays a very crucial role in the BI of elderly people in Saudi as a result of their unique cultural heritage.

The finding for H5 therefore, indicated that culture influence has a positive and significant effect on BI to use SHTs. In line with that is the finding by Barton [105]; Zhao & Khan [106]; Bankole and Bankole [107] who also found that culture influence is positively related to BI. There are four constructs of the UTAUT Model. Namely: performance expectancy, effort expectancy, social influence and facilitating conditioning. But this present study incorporates culture influence to further give an insight into the UTAUT model particularly in SHTs among elder people.

*Relationship between technology awareness and BI*. The sixth hypothesis (H6) of the study is whether technology awareness has a positive impact on BI to use SHTs. According to [63], understanding and awareness of technology are essential variables in adopting SHTs. A study [108] opined that one of the major challenges to smart home adoption has been identified as a lack of awareness of SHTs. The result of this research shows that technology awareness has a positive and significant effect on BI. The result of the study has previously been proved by [109, 110] who also found that there exists a positive and significant relationship between BI.

*Relationship between attitude and BI*. The seventh hypothesis (H7) of the study is whether attitude has a positive influence on BI to use SHTs. The study found a significant positive impact of attitude on BI to use SHTs, consistent with past studies [69, 70]. This present study confirms the importance of attitude in predicting BI to use SHTs among elderly people in Saudi Arabia because when the elderly have a positive attitude towards SHTs, they are more expected to increase their intention to use SHTs.

#### 5.3.2 Findings on the mediating hypotheses

In this section, the indirect relationship between constructs was discussed. The discussion of the mediating effect of attitude with, effort expectancy followed by performance expectancy was further explained.

*Mediating effect of attitude between effort expectancy and BI*. The study examined in hypothesis eight (8) the mediating effect of attitude on the relationship between effort expectancy and BI. The study found that attitude mediates the relationship between effort expectancy and BI among elderly people in Saudi Arabia to use SHTs. This finding is in line with the findings by [17, 24, 45].

The introduction of attitude as a mediating variable is to further explain the relationship between effort expectancy and BI. Studies have initially shown that there is a direct effect of effort expectancy on BI [99, 100, 111]. But this gives a holistic view through mediating variable (attitude) that ease of use is not the only significant determinant of the adoption of technology but also, attitude goes a long way to strengthen the given relationship.

*Mediating effect of attitude between performance expectancy and BI*. Under the indirect effect. The study examined in hypothesis nine (9) the mediating effect of attitude on the relationship between performance expectancy and BI. The study found that attitude mediates the relationship between performance expectancy and behavioural intention among elderly people in Saudi Arabia to use SHTs.

This result supports the finding by [24, 45]. Attitude plays a very crucial role in determining the BI to use SHTs. Though there are very limited studies on mediating role of attitude and this present study has established the fact that it also determines the BI for the elderly in Saudi Arabia to use SHTs. Pal, et al. [17] and Yang, et al. [45] carried out their studies in different contexts in South Korea and Finland respectively which can, therefore, make the generalization more realistic.

#### 5.3.3 Findings on the moderating hypotheses

In this section, the findings for the moderating relationship between the constructs were discussed. The discussion of the moderating effect of education and region on culture, technology awareness, and BI was supported by past research in technology adoption.

*The moderating role of education on culture influence and BI*. Hypothesis ten a (10a) stated that education moderates the relationship between culture influence and BI. The finding shows that education moderates the relationship between culture influence and behavioural intention to use SHTs in Saudi Arabia. Though, there may be a scarcity of literature on the moderating role of education on the relationship culture influence and BI. The present study has made itself a source of reference for any future research whether from the Arabian world or any other part of the continent.

Furthermore, a study by Mashal and Shuhaiber [2] can as well give an insight into the moderating role of education. This study shows that elderly people are more readily willing to accept and use SHTs once they are educated about their usefulness and benefits. The result further proved that culture cannot be the only determinant factor in behavioural intention to use SHTs among elderly people, but such desire can be strengthened through education.

*The moderating role of education on technology awareness and BI*. Hypothesis ten b (10b) stated that education moderates the relationship between technology awareness and BI. The finding shows that technology awareness moderates the relationship between culture influence and BI to use SHTs in Saudi Arabia. The findings of this study support Orlov [112] and Nikou [2] who also found that technology awareness moderates the relationship between culture influence and BI. There are limited studies so far on this, but this present study has been a milestone for future studies. Education and technology awareness almost play the same role, but their combination outcome has shown that apart from creating awareness of technology among the users.

There is still a need for how to apply it because this will make it user-friendly and encourage more people to show interest in it. The elderly people in Saudi usually stay indoors therefore, they may not have the opportunity to seek external assistance. Therefore, the role of education before using SHTs can never be underestimated.

*The moderating role of region on culture influence and BI*. Hypothesis eleven (11a) formulates that region moderates the relationship between culture influence and BI. The finding shows that the region does not moderate the relationship between culture influence and behavioural intention to use SHTs in Saudi Arabia. Although some studies [10, 105, 113] found culture influence to positively and significantly influence BI. this study does not confirm that region could moderate the relation between culture influence and BI. Hence, BI to use SHTs is not influenced by region as it does not play any major role regardless of its region.

*The moderating role of region on technology awareness and BI*. Hypothesis eleven b (11b) stated that region moderates the relationship between technology awareness and BI. The finding shows that region indeed moderates the relationship between technology awareness and BI to use SHTs in Saudi Arabia. This result supports the findings by [114, 115]. Therefore, as a result of limited studies on the role of region to moderate the relationship between technology awareness and BI, researchers should further explore the moderating role of region in smart home domains in other regions. Such results would establish whether there is consistency in the link between the level of technology awareness in regions that are more commercialized or business dominated.

#### 5.3.4 Practical contributions

Our findings have some practical and managerial implications. First, we establish that makers and marketers of SHTs must consider culture influence and technology awareness in their strategic decisions in selling SHTs applications and equipment. By considering these factors, the purchasing power and intention to use SHTs by elderly people may be enhanced. In addition, marketers of SHTs may focus on technology literacy programs in different healthcare premises and environments to enhance the level of SHTs awareness among elderly people.

For instance, SHTs manufacturers and suppliers may focus on the Western part (i.e., Jeddah) and the Central part (i.e., Riyadh), where a large percentage of businesses driving growth is centred. Elderly people in these regions are more likely to be aware of new technologies in the market and how such technologies such as SHTs could impact their lives positively. The results of our descriptive statistics confirm that the Western region, the Central Region, and the Eastern Region are more technologically advanced while the Southern and Northern regions are less technologically advanced. More than 80% of the elderly people sampled reside in technologically advanced regions (Western Region, Central Region, and Eastern Region). This further confirms the reason why the region moderates technology awareness but does not significantly moderate culture influence. The effect of this is not far-fetched. Elderly people may be more likely to adopt SHTs and monitors their daily health issues, which might reduce their health challenges in the long run and declines the amount of health-related costs associated with the non-use of SHTs. The government could also benefit from the findings of this study. As noted in the Saudi Vision2030 to ensure human capital development and improved standard of living, the government may also focus some of their attention and strategies on the provision of SHTs to elderly people.

#### 5.3.5 Theoretical contributions

Theoretically, we contribute to the existing literature in technology management. First, contextually, we advanced the UTAUT theory by adding two new factors; culture influence and technology awareness. Second, we also bridge the gap between the TAM model and UTAUT theory by adding attitude as a mediator factor in the UTAUT framework, by establishing that PE and EE could affect behavioural intention through elderly people’s attitude towards the SHTs. Our results confirm that elderly people in Saudi Arabia have a positive attitude towards SHTs. Moreover, we provide a novel moderator, region, as a contextual attribute rather than a demographic factor to enhance BI to use SHTs. We argue on the basis that elderly people and people in general in business and technological advanced regions are more expected to accept new technologies due to their level of awareness, technology, energy, and standard of living.

## 6. Conclusion

This study examines the determinants of BI to use SHTs among elderly people. This study from a contextual perspective extends the UTAUT theory using cultural influence and technology awareness. The study uses a survey approach to elicit responses from about 486 elderly people in Saudi Arabia.

The study finds that performance expectancy, effort expectancy, culture influence, technology awareness, and attitude have positive and significant effects on BI to use SHTs while social influence and facilitating conditions were negatively associated with BI to use SHTs. Our results of the mediating effect of attitude reveal that attitude mediates the relationships between performance expectancy, effort expectancy, and BI. Concerning the moderating hypotheses, this study finds considerable empirical evidence that education moderates the relationships between culture influence, technology awareness, and behavioural intention. However, we find mixed findings for region moderators. While region moderates the relationships between technology awareness on BI, it, however, fails to moderate the relationships between culture influence on BI to use SHTs.

The findings reveal that culture influence and technology awareness are important factors to determine BI to use SHTs in an Arab context. Thus, we generalize our findings to Saudi Arabia and other regions and countries where culture is a strong factor in decision-making. On the issue of technology awareness, this study’s findings on region suggest that in countries where certain regions are highly technological inclined, technology awareness is thus, a determining factor of behavioural intention to use smart home technologies.

Meanwhile, the purposive sampling technique adopted indicates that the findings of this study cannot be generalized to individuals who are less than 60 years. The limitation of this study is focusing on a certain age group e.g., elderly people. Future studies could focus on both young and elderly people to establish whether culture influence and technology awareness differ between these age groups. Lastly, future studies may further advance the UTAUT theory using factors such as training support, financial constraint, and third-party influence. These factors are potentials independent or moderating factors that can help enhance the BI to use SHTs and other related IoT technologies.
